# Clinical-learning versus machine-learning for transdiagnostic prediction of psychosis onset in individuals at-risk

**DOI:** 10.1038/s41398-019-0600-9

**Published:** 2019-10-17

**Authors:** Paolo Fusar-Poli, Dominic Stringer, Alice M. S. Durieux, Grazia Rutigliano, Ilaria Bonoldi, Andrea De Micheli, Daniel Stahl

**Affiliations:** 1 Early Psychosis: Interventions and Clinical-detection (EPIC) lab, Department of Psychosis Studies, Institute of Psychiatry, Psychology & Neuroscience, King’s College London, London, UK; 2 Department of Brain and Behavioural Sciences, University of Pavia, Pavia, Italy; 3 OASIS service, South London and Maudsley NHS Foundation Trust, London, UK; 4 National Institute of Health Research – Mental Health – Translational Research Collaboration – Early Psychosis Workstream, London, UK; 5 Department of Biostatistics and Health Informatics, Institute of Psychiatry, Psychology & Neuroscience, King’s College London, London, UK

**Keywords:** Schizophrenia, Diseases

## Abstract

Predicting the onset of psychosis in individuals at-risk is based on robust prognostic model building methods including a priori clinical knowledge (also termed clinical-learning) to preselect predictors or machine-learning methods to select predictors automatically. To date, there is no empirical research comparing the prognostic accuracy of these two methods for the prediction of psychosis onset. In a first experiment, no improved performance was observed when machine-learning methods (LASSO and RIDGE) were applied—using the same predictors—to an individualised, transdiagnostic, clinically based, risk calculator previously developed on the basis of clinical-learning (predictors: age, gender, age by gender, ethnicity, ICD-10 diagnostic spectrum), and externally validated twice. In a second experiment, two refined versions of the published model which expanded the granularity of the ICD-10 diagnosis were introduced: ICD-10 diagnostic categories and ICD-10 diagnostic subdivisions. Although these refined versions showed an increase in apparent performance, their external performance was similar to the original model. In a third experiment, the three refined models were analysed under machine-learning and clinical-learning with a variable event per variable ratio (EPV). The best performing model under low EPVs was obtained through machine-learning approaches. The development of prognostic models on the basis of a priori clinical knowledge, large samples and adequate events per variable is a robust clinical prediction method to forecast psychosis onset in patients at-risk, and is comparable to machine-learning methods, which are more difficult to interpret and implement. Machine-learning methods should be preferred for high dimensional data when no a priori knowledge is available.

## Introduction

Under standard care, outcomes of psychosis are poor^1^. While early interventions at the time of a first psychotic episode are associated with some clinical benefits^2^, they are not effective at preventing relapses^2^ or reducing the duration of untreated psychosis (DUP)^3^; preventive interventions in individuals at clinical high risk for psychosis (CHR-P)^4^ may be an effective complementary strategy. According to the World Health Organization, preventive strategies for mental disorders are based on the classification of the prevention of physical illness as universal, selective or indicated (targeted at the general public, those with risk factors, and those with minimal signs or symptoms of mental disorders respectively, as described by by Gordon et al.) and on the classic public health classification as primary, secondary or tertiary (seeking to prevent the onset of a mental disorder, lower the rate of established disorder or reduce disability and relapses, respectively^5^). Universal, selective and indicated preventive interventions are “included within primary prevention in the public health classification” (page 17 in ref. ^5^). Since CHR-P individuals show attenuated symptoms of psychosis coupled with help-seeking behaviour^6^ and functional impairments^7^, interventions in these individuals are defined as indicated primary prevention of psychosis. The conceptual and operational framework that characterises the CHR-P paradigm has been reviewed elsewhere^8,9^. The empirical success of the CHR-P paradigm is determined by the concurrent integration of three core components: efficient detection of cases at-risk, accurate prognosis and effective preventive treatment^10,11^. The underpinning methodology for each of these components is based on risk-prediction models^12^. Unfortunately, a recent methodological review concluded that most of the CHR-P prediction modelling studies are of low quality, largely because they employ stepwise variable selection without proper internal and external validation^13^. These approaches overfit the data (i.e. the model learns the noise instead of accurately predicting unseen data^14^), inflate the estimated prediction performance on new cases and produce biased prognostic models that result in poor clinical utility^14^. Beyond stepwise model selection, overfitting can also occur when the number of events (e.g. number of at-risk patients who will develop psychosis over time) per variable (e.g. degree of freedoms of predictors of psychosis onset in at-risk patients) is low (event-per-variable, EPV <20^14,15^). Low EPVs are frequently encountered in the CHR-P literature because the onset of psychosis in these samples is an infrequent, heterogeneous event (cumulating to 20% at 2-years, (eTable 4 in ref. ^16^; depending on the sampling strategies)^17–20^.

A first approach to overcome these caveats is to use a priori clinical-learning or knowledge to identify a few robust predictors to be used in risk-prediction models^13^: it may be possible to use umbrella reviews (i.e. reviews of meta-analyses and systematic reviews that incorporate a stratification of the evidence^21^) on epidemiological risk/protective factors for psychosis^22^). Because the selection of predictors would be limited in number (preserving the EPV^14^) and independent of the data on which the model is then tested, overfitting issues would be minimised^13^. For example, a recent risk estimation model has used a priori clinical-learning to select a few predictors of psychosis onset in CHR-P individuals^23^. The prognostic model developed was robust and has already received several independent external replications^24^. A second, increasingly popular approach is to bypass any clinical reasoning and instead use machine-learning procedures to select the predictors automatically^25^: machine-learning studies have developed and internally validated models to stratify risk enrichment in individuals undergoing CHR-P assessment^18^ and functional outcomes in CHR-P samples^26^. Machine-learning methods promise much to the CHR-P field because of their potential to assess a large number of predictors and to better capture non-linearities and interactions in data; there is great confidence that they will outperform model-building based on clinical learning^25^. Yet, modern machine-learning methods may not a panacea^27^, particularly because of the lack of empirical research comparing machine-learning vs clinical-learning theory-driven methods for the prediction of psychosis. The current manuscript advances knowledge by filling this gap.

Here we use a transdiagnostic, prognostic model that has been developed by our group using a priori meta-analytical clinical knowledge (hereafter clinical-learning)^28^. The predictors used were collected as part of the clinical routine: age, gender, ethnicity, age by gender and ICD-10 index diagnostic spectrum. The model is cheap and “transdiagnostic”^29^ because it can be applied at scale across several ICD-10 index diagnoses to automatically screen mental health trusts. This prognostic model has been externally validated twice^28,30^, and is under pilot testing for real-world clinical use^11^.

In the first experiment, we apply a machine-learning method to the same transdiagnostic individualised prognostic model and test the hypothesis that machine-learning methods produce models with better prediction accuracy than clinical-learning approach when the EPV is adequate. In the second experiment, we expand the granularity of the ICD-10 index diagnosis predictor and test the hypothesis that the use of more specific diagnostic specifications improves prognostic performance. In the third experiment, we test the hypothesis that machine-learning delivers better predicting prognostic models than clinical-learning under different models’ specifications, and in the specific scenario of low EPVs. Overall, this study provides much needed empirical research to guide prediction modelling strategies in early psychosis.

## Materials and methods

### Data source

Clinical register-based cohort selected through a Clinical Record Interactive Search (CRIS) tool^31^.

### Study population

All individuals accessing South London and Maudsley (SLaM) services in the period 1 January 2008–31 December 2015, and who received a first ICD-10 index primary diagnosis of any non-organic and non-psychotic mental disorder (with the exception of Acute and Transient Psychotic Disorders, ATPDs) or a CHR-P designation (which is available in the whole SLaM through the Outreach And Support In South-London -OASIS- CHR-P service^32^), were initially considered eligible. The ATPD group is diagnostically^33^ and prognostically^34^ similar to the Brief Limited Intermittent Psychotic Symptom (BLIPS) subgroup of the ARMS construct and to the Brief Limited Psychotic Symptoms (BIPS) subgroup of the Structured Interview for the Psychosis-Risk Syndrome (SIPS; for details on these competing operationalisation see eTable 1 published in ref. ^34^) and previous publications on the diagnostic and prognostic significance of short-lived psychotic disorders^33,35,36^.

Those who developed psychosis in the three months immediately following the first index diagnosis were excluded. As previously detailed, this lag period was chosen to allow patients sufficient time after their index diagnosis to meet the ICD-10 duration criterion for ATPDs. Since we did not employ a structured assessment at baseline (see limitation), this lag period was also used to be conservative and exclude individuals who were underreporting psychotic symptoms at baseline (false transition to psychosis).

Ethical approval for the study was granted^31^.

### Study measures

The outcome (risk of developing any ICD-10 non-organic psychotic disorder), predictors (index ICD-10 diagnostic spectrum, age, gender, ethnicity, and age by gender), and time to event were automatically extracted using CRIS^31^.

### Statistical analyses

The original study was conducted according to the REporting of studies Conducted using Observational Routinely-collected health Data (RECORD) Statement^37^.

#### Experiment 1: Machine-learning vs clinical-learning with adequate EPV for the prediction of psychosis

Development and validation of the original model (M1, diagnostic spectra) followed the guidelines of Royston et al.^38^, Steyerberg et al.^39^ and the Transparent Reporting of a multivariable prediction model for Individual Prognosis Or Diagnosis (TRIPOD)^40^. The details of model development and external validation have been presented previously^28^. Briefly, predictors (ICD-10 diagnostic spectrum, age, gender, ethnicity, and age by gender interaction) were preselected on the basis of meta-analytical clinical knowledge^41^ as recommended^13^. The ICD-10 diagnostic spectrum was defined by all of the ten ICD-10 blocks (acute and transient psychotic disorders, substance abuse disorders, bipolar mood disorders, non-bipolar mood disorders, anxiety disorders, personality disorders, developmental disorders, childhood/adolescence onset disorders, physiological syndromes and mental retardation^28^), with the exclusion of psychotic and organic mental disorders, and by CHR-P designation^8^. Accordingly, the diagnostic predictor of M1 encompassed 11 different levels. All other predictors together contributed 7 degrees of freedom, for a total of 18 degrees of freedom. Cox proportional hazards multivariable complete-case analyses were used to evaluate the effects of preselected predictors on the development of non-organic ICD-10 psychotic disorders, and time to development of psychosis. Non-random split-sample by geographical location was used to create a development and external validation dataset^40^. Performance diagnostics of individual predictor variables in the derivation dataset were explored with Harrell’s C-index^38^, which can be interpreted as a summary measure of the areas under the time-dependent ROC curve^42^. A value of *C* = 0.5 corresponds to a purely random prediction whereas *C* = 1 corresponds to perfect prediction. The model was then externally validated in the independent database from SLaM^28^, and subsequently in another NHS Trust (Camden and Islington)^30^. In the SLaM derivation database there were 1001 events (EPV 1001/18 = 55.61), and in the SLaM validation database there were 1010 events, both of which exceed the cut-off of 100 events required for reliable external validation studies^43^.

In experiment 1, we tested the hypothesis that even when EPVs are above the recommended threshold and predictors are the same, machine-learning would outperform clinical-learning methods. Machine learning methods automate model building by learning from data with minimal human intervention^44^; the best model is typically selected by assessing the prediction accuracy of unseen (hold-out) data for example using cross-validation methods^45^. This is a key difference from classical statistical inferential methods, where the quality of a model is assessed by the sample used to estimate the model. Machine-learning methods typically introduce a regularisation term into the model to avoid overfitting, and this term usually imposes a penalty on complex models to reduce sample variance^45^.

In our study we used regularised regression methods (also called penalised or shrinkage regression methods) as relatively simple, but often powerful machine learning methods which compare competitively to more complex machine learning methods like random forest or support vector machines^46–48^. We chose regularised regression methods to enhance interpretability of the final model, in particular compared to models developed through clinical learning. It is important for clinicians to interpret prognostic models to gain knowledge and to detect their potential biases and limitations in real-world use^49^. Regularised regression fits generalised linear models, for which the sizes of the coefficients are constrained to reduce overfitting. Two common regularised regression approaches to be considered in this study are RIDGE^50^ and LASSO^51^. The primary difference between RIDGE and LASSO is that RIDGE regression constrains the sum of squares of the coefficients, whereas LASSO constrains the sum of absolute values of the coefficients^45^. Unlike RIDGE, LASSO shrinks the coefficient to zero and thus performs an automatic selection of predictors. The degree of constraint (or penalty) is determined by automated computer-intensive grid searches of tuning parameters. Because constraints depend on the magnitude of each variable, it is necessary to standardise variables. The final tuning parameter is chosen as the one which maximises a measure of prediction accuracy of unseen (hold-out) data using, for example, cross-validation methods^45^.

Therefore, in experiment 1, we applied RIDGE and LASSO to the original unregularized Cox regression model in the same database to estimate their apparent and external performance (Harrell’s C) in the derivation and validation datasets respectively. Their difference was then used to estimate the model’s optimism.

#### Experiment 2: Diagnostic subdivisions vs diagnostic categories vs diagnostic spectra for the prediction of psychosis

We developed two refined prognostic models, M2 and M3, which differed from the original M1 model (diagnostic spectra, e.g. F30-F39 Mood [affective] disorders) by employing two expanded definitions of the predictor ICD-10 index diagnosis (the strongest predictor of the model^28,30^). The model M2 (diagnostic categories) expanded the M1 model by adopting the 62 ICD-10 diagnostic categories—excluding psychotic and organic mental disorders—rather than the broader spectra (e.g. F30 manic episode, F31 Bipolar affective disorders etc.). The model M3 (diagnostic subdivisions) further expanded the M2 model by including all of the 383 specific ICD-10 diagnostic subdivisions of non-organic and non-psychotic mental disorder (e.g. F30.0 hypomania, F30.1 mania without psychotic symptoms, F30.2 mania with psychotic symptoms, F30.8 other manic episodes, F30.9 manic episode unspecified). From a clinical point of view, these refined models reflect the potential utility of specific vs block vs spectrum diagnostic formulations for the prediction of psychosis onset in at-risk individuals. The two previous independent replications of the original M1 model confirmed that the clinicians’ pattern recognition of key diagnostic spectra is useful from a clinical prediction point of view. Thus, experiment 2a tested the clinical hypothesis that the use of more granular and specific ICD-10 index diagnoses would eventually improve the performance of the initial M1 model. The performance of the M1, M2 and M3 models was first reported in the derivation and validation dataset. In a subsequent stage, the model’s performance (Harrell’s C) was compared across each pair within the external validation dataset.

#### Experiment 3a and 3b. Machine-learning vs clinical-learning under variable EPVs

From a statistical point of view, increasing the number of levels of the ICD-10 diagnoses from M1 (*n* = 10) to M2 (*n* = 62) to M3 (*N* = 383) (plus the CHR designation), decreases the EPV from M1 to M2 to M3 respectively, increasing the risk of overfitting in unregularised regression models (in particular when the EPV is lower than 20^52^).

During experiment 3a, we tested the hypothesis that machine-learning would increasingly outperform clinician learning methods with decreasing EPVs. First, we compared the apparent performance of M1, M2, M3 in the whole dataset using RIDGE and LASSO versus unregularised Cox regression. Second, we compared the internal performance of M1, M2 and M3 in the whole dataset using ten-fold cross-validation repeated 100 times and taking the median Harrell’s C across the 100 repetitions, again using RIDGE, LASSO versus unregularized Cox regression. We used the whole dataset because the refined M2 and M3 models have adopted different specifications of the ICD-10 diagnoses that were not always present in both derivation and validation datasets (in which case it would not have been possible to test the same model). In the light of the decreased EPVs we expected RIDGE and LASSO to perform better for M3 than for M2 than for M1, respectively^45^).

In experiment 3b, we further assessed the impact of varied sample size and degree of EPV on the prognostic performance of the model M1 under machine-learning vs clinical-learning, without the confounding effect of including more potentially informative predictors. We randomly selected samples of different sizes from the derivation dataset and then fitted the machine-learning vs clinical-learning approaches to these samples. We then assessed the prediction accuracy in the external validation dataset. For each sample size, the results of ten repetitions with different random samples were averaged, and the median Harrell’s C reported for both the derivation (apparent) and validation datasets. Samples sizes were 500, 1000, 2000 and 5000.

All analyses were conducted in STATA 14 and R 3.3.0. using the user-written R packages “Coxnet” for the regularised Cox regression models and “Hmisc” to calculate Harrell’s C. The difference between two C’s were calculated using the STATA package “Somersd” and the R package “Rms”. Compute code is available from the authors (DS) upon request.

## Results

### Sociodemographic and clinical characteristics of the sample

91199 patients receiving a first index diagnosis of non-organic and non-psychotic mental disorder within SLaM in the period 2008–2015 fulfilled the study inclusion criteria and were included in the derivation (33820) or validation (54716) datasets. The baseline characteristics of the study population, as well as the derivation and validation datasets, are presented in Table 1^28^. The mean follow-up was 1588 days (95% CI 1582–1595) with no significant differences between the derivation and validation datasets.

**Table 1:** Sociodemographic characteristics of the study population, including the derivation and validation dataset^28^

	Derivation dataset		Validation dataset	
	Mean	SD	Mean	SD
Age (years)^c^	34.4	18.92	31.98	18.54
	Count	%	Count	%
Gender				
Male	17303	48.81	27302	49.9
Female	16507	51.16	27398	50.07
Missing	10	0.03	16	0.03
Ethnicity				
Black	6879	20.34	7023	12.84
White	18627	55.08	35392	64.68
Asian	1129	3.34	2608	4.77
Mixed	1306	3.86	1957	3.58
Other	3466	10.25	2084	3.81
Missing	2413	7.13	5652	10.33
ICD-10 Index spectrum diagnosis				
CHR-P^a^	314	0.93	50	0.09
Acute and transient psychotic disorders	553	1.64	725	1.33
Substance use disorders	7149	21.14	6507	11.89
Bipolar mood disorders	950	2.81	1526	2.79
Non-bipolar mood disorders	6302	18.63	8841	16.16
Anxiety disorders	8235	24.35	15960	29.17
Personality disorders	1286	3.8	2116	3.87
Developmental disorders	1412	4.18	3706	6.77
Childhood/adolescence onset disorders	4200	12.42	9629	17.6
Physiological syndromes	2555	7.55	4424	8.09
Mental retardation	864	2.55	1232	2.25

^a^Lambeth and Southwark, *n* = 33820

^b^Croydon and Lewisham, *n* = 54716

^c^Not an ICD-10 Index spectrum diagnosis

#### Experiment 1: Machine-learning vs clinical-learning and adequate EPV for the prediction of psychosis

The first analysis compared M1 model performance developed with clinician learning (a priori knowledge) against RIDGE and LASSO in both the derivation and validation dataset. Harrell’s C on derivation set was virtually the same for all three methods on both derivation (~0.8) and external validation data sets (~0.79, Table 2).

**Table 2:** Experiment 1: prognostic accuracy (Harrell’s C) for the original model (M1, diagnostic spectra) developed through Clinical-learning (a priori clinical knowledge) vs machine learning (LASSO and RIDGE). The EPV is >20 (55.6)

Method	Derivation Data Set (*N* = 33,820)			Validation Data Set (*N* = 54,716)			Optimism
	Harrell’s C	SE	95% C.I.	Harrell’s C	SE	95% C.I.	
Unregularized	0.800	0.008	0.784–0.816	0.791	0.008	0.775–0.807	0.009
Lasso	0.798	0.008	0.782–0.814	0.789	0.008	0.773–0.805	0.009
Ridge	0.810	0.008	0.794–0.826	0.788	0.008	0.772–0.804	0.022

#### Experiment 2: diagnostic subdivisions vs diagnostic categories vs diagnostic spectra for the prediction of psychosis

The database included the majority of the non-organic and non-psychotic ICD-10 diagnostic categories (57 out of 62, 92% in M2), and diagnostic subdivisions (353 out of 383, 92% in M2).

In the derivation dataset (apparent performance^14^), the M3 model (Harrell’s C 0.833) seemed to perform better, than the M2 model (Harrell’s C 0.811) and better than the original M1 model (Harrell’s C 0.8). However, this was due to overfitting of the M3 to the derivation data, as confirmed by the external validation. In fact, in the validation dataset, using all of the ICD-10 diagnostic subdivisions (M3) yielded a comparable model performance (about 0.79) to M1 and comparable to the model with the diagnostic categories (M2). The latter model (M2) showed statistically significant, superior performance compared to M1. However, the magnitude of the improvement of the Harrell’s C of 0.007 was too small to be associated with meaningful clinical benefits (see Table 3).

**Table 3:** Experiment 2: prognostic performance of the revised models in the derivation dataset and the validation dataset, and their comparative performance

Model	Type of clustering of ICD-10 index diagnoses	Harrell’s C	SE	95% CI	
Derivation dataset					
M1	Diagnostic spectra	0.800	0.008	0.784	0.816
M2	Diagnostic categories	0.811	0.008	0.795	0.824
M3	Diagnostic subdivisions	0.833	0.008	0.821	0.847
Validation dataset					
M1	Diagnostic spectra	0.791	0.008	0.776	0.807
M2	Diagnostic categories	0.797	0.008	0.782	0.812
M3	Diagnostic subdivisions	0.792	0.008	0.776	0.808
M2-M1		0.006	0.003	0.001	0.012
M3-M1		0.001	0.005	−0.009	0.011
M3-M2		−0.005	0.005	−0.015	0.004

All models include age, gender, age by gender, ethnicity and ICD-10 index diagnosis (refined as specified in the methods)

#### Experiment 3a and 3b. Prognostic performance using machine-learning vs clinical-learning under variable EPVs

The results from experiment 3a showed that the clinical-learning and machine-learning methods delivered similar apparent prognostic performance (Table 4). After internal validation, Harrell’s C slightly decreased, and M1, M2 and M3 models were all similar (approximately 0.8). There were again small differences between clinical-learning and machine-learning methods, which were more marked as EPV decreased.

**Table 4:** Experiment 3a. Prognostic performance using machine-learning vs clinical-learning under variable EPVs

	Unregularized								
	M1 (diagnostic spectra)			M2 (diagnostic categories)			M3 (diagnostic subdivisions)		
	Cox Regression	LASSO	RIDGE	Cox Regression	LASSO	RIDGE	Cox Regression	LASSO	RIDGE
Apparent performance									
C index	0.800	0.793	0.790	0.811	0.799	0.803	0.827	0.812	0.813
SE	0.005	0.005	0.006	0.005	0.005	0.005	0.005	0.005	0.005
Internal validation performance									
C index	0.799	0.794	0.790	0.804	0.795	0.795	0.805	0.793	0.797
SE	0.017	0.017	0.017	0.017	0.017	0.017	0.017	0.017	0.017
Events	2011			2011			2011		
Degrees of freedom of predictors	18			63			226		
EPV	111.7			31.9			8.9		

Upper part of the table: apparent performance of M1-M3 models in the whole dataset. Bottom part of the table: internal performance in the whole dataset using nested 10-fold CV and taking median values with 100 repetitions

EPV events per variables, calculated as the number of transitions to psychosis over the degrees of freedom of predictors. Categorical predictors are counted as the number of indicator categories they consist of (i.e. number of categories−7)

**Fig. 1:**
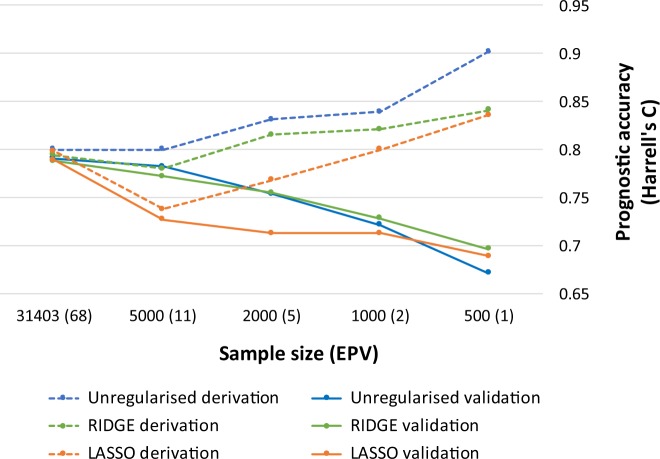
Experiment 3b. Clinical-learning (unregularised regression) vs machine learning (LASSO and RIDGE) for the original model M1 with random sampling of varying sample sizes and decreasing EPV

In experiment 3b, Harrell’s C for M1 in the derivation dataset increased with decreasing sample size. The increase was larger for clinical-learning (unregularized regression: from 0.8 to 0.9), and smaller for machine-learning (RIDGE and LASSO: 0.79–0.83, Fig. 1). The opposite pattern was then seen in the external validation dataset, where Harrell’s C for M1 decreased with decreasing sample size. Hence, optimism (the difference between Harrell’s C in the apparent sample and with internal validation) increased with smaller sizes. As sample size decreased, Harrell’s C decreased slightly more when using clinical-learning (unregularized regression: from 0.79 to 0.67 if *N* = 500) than when using machine-learning (RIDGE regression: from 0.79 to 0.70 and LASSO regression: from 0.79 to 0.69).

## Discussion

This study compared clinical-learning vs machine-learning methods for the prediction of individuals at-risk for psychosis. The first experiment indicated that clinical-learning methods with a priori selection of predictors and adequate EPV produce robust prognostic models that are comparable to those obtained through regularised regression machine-learning methods. The second experiment indicated that there is no improvement in prognostic accuracy when specific ICD-10 diagnoses are employed instead of broad diagnostic spectra. The third experiment indicated that machine learning methods can deliver more robust prognostic models that clinical-learning methods when the sample size is small and the EPV low, although the benefits are modest in magnitude.

The first hypothesis of the current study was that machine-learning methods would generally outperform clinical-learning methods using the same set of predictors. This was not verified in our study, because when RIDGE and LASSO methods were applied to the previously published transdiagnostic individualised risk estimation model, there was no substantial difference in prognostic performance. This suggests that when a prognostic model is built on strong clinical knowledge, has a large sample and an adequate EPV (in this case it was 56), the model can perform very well without the use of machine-learning methods. Machine-learning methods are not always necessary to obtain an accurate prediction of psychosis onset and do not necessarily improve the performance of prognostic models developed on a priori clinical knowledge. For example, a recently published supervised machine-learning study failed to demonstrate improved prediction of transition to psychosis when using baseline clinical information with no a priori knowledge^53^, suggesting that a priori clinical knowledge remains very important for developing good prognostic models. Given a comparable accuracy, models developed through clinical-learning tend to be more straightforward and thus more likely to be interpreted, assessed and accepted, and implemented in clinical care (see below).

Our second hypothesis was that adding more information to the model by expanding the granularity of the ICD-10 index diagnosis would improve prognostic performance. The results showed no prognostic benefit to using specific ICD-10 diagnoses compared to broad diagnostic spectra for the prediction of psychosis in secondary mental health care. The diagnostic spectra employed by the original version of the transdiagnostic individualised risk calculator^28^ are robust because they originate in prototypical descriptions containing a core phenomenological structure (gestalt) of the disorder and its polysymptomatic manifestations^29^. Examination of overlaps of etiological factors between disorders confirms that higher level broad diagnostic constructs may be more valid and clinically useful categories than specific diagnostic categories^54^. The prognostic utility of the ICD-10 diagnostic spectra is also in line with recent meta-analytical findings indicating that diagnostic spectra (e.g. psychosis) are relatively stable at the time of a first episode of psychosis^55^. These diagnostic spectra are certainly not optimal, yet they do not present an insuperable barrier to scientific progress^56^, and in terms of scalability in secondary mental health care^57^ have yet to be beaten by other predictors of psychosis onset. Conversely, available clinical evidence indicates that the specific ICD-10 diagnoses are unreliable and unstable, and this may explain why their use is associated with overfitting problems and lack of prognostic benefits^55^. It is also possible that the small number of cases observed in some specific diagnostic categories may interfere with the efficacy of machine learning approaches.

The third hypothesis was that LASSO and RIDGE would perform better in the presence of either unstable (such as the specific ICD-10 diagnoses) or redundant predictors, or infrequent events (low EPV); RIDGE is generally better with a small number of unstable predictors, and LASSO with a large number. This hypothesis was confirmed: the best performing model under low EPV and unstable predictors was obtained through machine-learning approaches^13^. However, the improvement in prognostic performance was modest, indicating that if strong predictors are known in advance through clinical-learning, it may be difficult to improve the model by adding many other variables which are more likely to be interpreted as noise, even when using penalized regression machine-learning methods. Notably, our study tested only two simple machine learning methods (RIDGE and LASSO), so we cannot exclude the possibility that prognostic improvements may have been larger if more complex machine learning methods (such as random forest or support vector machines for survival) have been used^58,59^. However, Ploeg, Austin, and Steyerberg demonstrated that the development of robust models by machine-learning methods requires more cases-per-candidate predictors than traditional statistical methods when the dimensionality is not extremely high^27^. Interestingly, even if large data sets are available, complex machine learning methods (i.e. random forests) only showed only minor improvement (at the expense of reduced interpretability and no automatic variable selection) over simple statistical models^27^. This view was pragmatically supported by a recent systematic review which compared random forests, artificial neural networks, and support vector machines models to logistic regression. Across 282 comparisons, there was no evidence of superior performance of machine-over clinical-learning for clinical prediction modelling^60^.

Not surprisingly, the prognostic tools used to date in the real world clinical routine of CHR-P services are still based on clinical-learning^23,28^. However, in the current study, we could not test whether the addition of new multimodal predictors - beyond the clinical and sociodemographic ones—would improve the prognostic accuracy of psychosis onset. Some studies have suggested that the combination of clinical information with structural neuroimaging measures (such as gyrification and subcortical volumes) could improve prognostic accuracy^61^. However, available studies failed to provide convincing evidence that multimodal predictors under machine learning can substantially improve prognostic accuracy for predicting psychosis onset in patients at risk^62,63^. Furthermore, complex models based on multimodal domains are constrained by logistical and financial challenges that can impede the ability to implement and scale these models in the real world. A potentially promising solution may be to adopt a sequential testing assessment to enrich the risk in a stepped framework, as demonstrated by our group with a simulation meta-analysis^64^. Interestingly, a recent machine-learning study on patients at-risk for psychosis confirmed that adding neuroimaging predictors to clinical predictors produced a 1.9-fold increase in prognostic certainty in uncertain cases of patients at-risk for psychosis^26^.

Our study provides some conceptual and broad implications; although machine learning methods have attracted high expectations in the field^25,65,66^, the enthusiasm may not be entirely substantiated in the field of psychosis. First, we have demonstrated that if robust a priori clinical knowledge is available, and if there are large sample sizes and EPVs, clinical-learning is a valid method to develop robust prognostic models. Clearly, a priori clinical knowledge may not always be available, and high dimensional databases with large sample sizes or strong signal to noise ratio may be needed to address the complexity of mental disorders. Under those circumstances, machine-learning methods can produce more robust prognostic models. Our study also provides support for this situation where detailed clinical information is not available; machine learning methods were able to identify models of similar prediction accuracy.

Second, the methodological, empirical and conceptual limitations of machine learning in psychiatry have not been completely addressed. Overoptimistic views, excessive faith in technology^67^ and lack of knowledge of limitations of a specific methodology can lead to unrealisable promises^68^. While machine learning methods can potentially achieve good predictive accuracy in high dimensional data when there is poor a priori knowledge, they tend to deliver “black-box” classifiers that provide very limited explanatory insights into psychosis onset^69^. This is a fundamental limitation: without direct interpretability of a prognostic procedure, implementation in clinical practice may be limited^68^. To have high impact and be adopted on a broader scale, a prognostic model must be accepted and understood by clinicians. Prediction models developed through clinical-learning are traditionally better understood by clinicians than machine learning models^70^, while machine-learning models are challenging to evaluate and apply without a basic understanding of the underlying logic on which they are based^71^. A partial solution may be to incorporate a priori knowledge into machine-learning approaches^72^. Because of these issues, some authors argue that clinical-learning and reasoning will become even more critical to distil machine-learning and data-driven knowledge^73^, and preliminary studies suggest that the combined use of theory-driven and machine learning approaches can be advantageous^74^. There is a trend towards converting “big data” into “smart data” through contextual and personalised processing, allowing clinicians and stakeholders to make better decisions; our study supports such an approach^75^.

Third, an additional pragmatic limitation is that for prediction models to ultimately prove useful, they must demonstrate impact^76^—their use must generate better patient outcomes^70^. Impact studies for machine-learning approaches in patients at-risk for psychosis are lacking. Rigorous tests on independent cohorts are critical requirements for the translation of machine-learning research to clinical applications^77^. To our knowledge, the only study that has estimated the potential clinical benefit associated with the use of a prognostic model in secondary mental health care is our transdiagnostic individualised risk calculator analysis, which was based on clinical-learning^28^. A recent review observed that although there are thousands of papers applying machine-learning algorithms to medical data, very few have contributed meaningfully to clinical care^78^. Another recent empirical study focusing on the clinical impact of machine-learning in early psychosis concluded that the current evidence for the diagnostic value of these methods and structural neuroimaging should be reconsidered toward a more cautious interpretation^79^.

## Conclusions

Developing prognostic models on the basis of a priori clinical knowledge, large samples and adequate events per variable is a robust clinical prediction method for forecasting psychosis onset in patients at-risk. Under these circumstances, the prognostic accuracy is comparable to that obtained through machine-learning methods, which are more difficult to interpret and may present additional implementation challenges. The use of diagnostic spectra for transdiagnostic prediction of psychosis in secondary mental health care offers superior prognostic accuracy than the use of more specific diagnostic categories. Machine-learning methods should be considered in cases of high dimensional data when no a priori knowledge is available.
